# Molecular-Level Structure Regulation via Sequential Pre-Crosslinking and Friedel-Crafts Hypercrosslinking: Fabrication of Bimetallic Doped Porous Carbon Electrocatalysts

**DOI:** 10.3390/molecules31193510

**Published:** 2026-10-01

**Authors:** Yang Du, Shuo Gao, Jinyuan Zhang

**Affiliations:** 1 Institute of Petrochemical Technology, Jilin University of Chemical Technology, NO. 45 Chengde Street, Jilin 132022, China; 2 School of Chemical and Pharmaceutical Engineering, Jilin University of Chemical Technology, NO. 45 Chengde Street, Jilin 132022, China

**Keywords:** polystyrene, hypercrosslinking, porous carbon, alkaline OER

## Abstract

Owing to high surface inertness and insufficient active sites, traditional carbons cannot anchor and disperse metal species evenly, restricting catalytic activity. Via polymer precursor design, the components, functional groups and pore architecture can be tailored molecularly and preserved upon carbonization, thus regulating carbon microstructure, surface properties and active centers efficiently. This work focuses on the synthesis and structural tuning of polystyrene-based precursors using a three-step procedure of pre-crosslinking, Friedel-Crafts hypercrosslinking, and activation. The materials possess abundant mesopores ranging from 7 to 30 nm, which serve as an ideal platform for the efficient anchoring of metal ions and mass transfer during the subsequent carbonization and functionalization processes. Based on the strong adsorption and exchange ability of sulfonic acid groups, transition metal ions such as Co, Ni and Fe were uniformly doped on the surface of nanoparticles and carbonized at 300–600 °C in an air atmosphere to realize the conversion of the polymer skeleton to a carbon matrix and the in situ formation of metal species into oxide nanoparticles. At the application performance level, the prepared Co/Ni bimetallic-carbon-doped material exhibits excellent electrocatalytic activity in the alkaline oxygen evolution reaction. The overpotential is only 0.3478 V at a current density of 10 mA cm^−2^. After 50 cycles of cyclic voltammetry, the voltage fluctuation is only 0.026 V.

## 1. Introduction

Porous carbon materials with ultrahigh specific surface area offer abundant active sites and adsorption channels and enable efficient charge transfer owing to excellent electrical conductivity [1,2,3,4,5]. Moreover, they possess excellent chemical stability and can maintain structural integrity even under harsh and complex reaction conditions [6,7,8]. For these merits, porous carbons have been extensively applied in numerous important fields including heavy metal ion removal, organic pollutant adsorption, and catalytic conversion (e.g., electrocatalysis and photocatalysis), and energy storage (e.g., electrode materials for supercapacitors and lithium-ion batteries) [9,10,11,12,13,14]. Nevertheless, carbon substrates suffer from inherent surface inertness, cumbersome post-modification procedures, and insufficient functional design, which limit their practical application.

Various methods, including direct carbonization, activation and templating, have been established to tailor the pore structure of porous carbons through physical and chemical means [15,16,17]. Direct carbonization refers to the preparation route in which carbon-containing precursors are subjected to high-temperature thermal treatment under an inert atmosphere. Pore structures are spontaneously generated owing to the intrinsic architecture of precursors, the escape of endogenous small-molecule gases during pyrolysis, and thermal shrinkage of the carbon skeleton.

Nevertheless, this method is greatly restricted by the physicochemical properties of raw precursors. In this context, polymer-derived carbon materials represent a favorable alternative, as they possess the distinctive advantage of precursor engineering. Through precise modulation ranging from molecular structural design to macroscopic morphology, the pre-designed structural features of polymer precursors can be inherited by the final carbon products during carbonization, thereby enabling the directional regulation of the structure and performance of as-obtained materials [18,19,20]. For this reason, polymer-derived carbons serve as an ideal platform for fabricating functional carbon materials. They exhibit exceptional performance in application scenarios with stringent structural requirements such as high-efficiency electrocatalysis, and have become a prominent research hotspot in this field to date [21,22].

Polymer-derived carbon materials possess tunable porous architectures and allow facile heteroatom doping and metal functionalization, rendering them widely applicable in electrocatalytic oxygen evolution reactions [23,24]. Polystyrene-based precursors have garnered substantial research interest in recent years by virtue of their good moldability, easy functional modification, and controllable pyrolysis behavior [25,26]. For polystyrene-based precursor fabrication, the choice of polymerization procedure is critical, as it directly dictates the precursor’s molecular-weight distribution, microscopic morphology, cross-linking density, and extent of functional-group modification. These properties in turn define the pore features, active-site distribution, and electrocatalytic performance of the final carbonized products.

Polystyrene is intrinsically a linear polymer. Despite its high carbon content, carbon materials obtained via direct carbonization of pure PS tend to undergo melting and decomposition upon heating, which results in low carbonization yield, collapsed microstructure and unsatisfactory electrocatalytic activity. Physical or chemical modification strategies are required to construct rigid carbon skeletons, improve thermal stability and carbonization yield. Typically, chemical crosslinking is adopted to construct a three-dimensional network structure for polystyrene and enhance its thermal stability as a carbon precursor [27,28,29]. Sulfonation modification is utilized to endow precursors with strong cation-exchange capacity, facilitating the efficient immobilization of various metal ions [30,31]. Moreover, such modification introduces heteroatoms into the matrix, which further creates abundant catalytic active sites.

**Figure 1:**
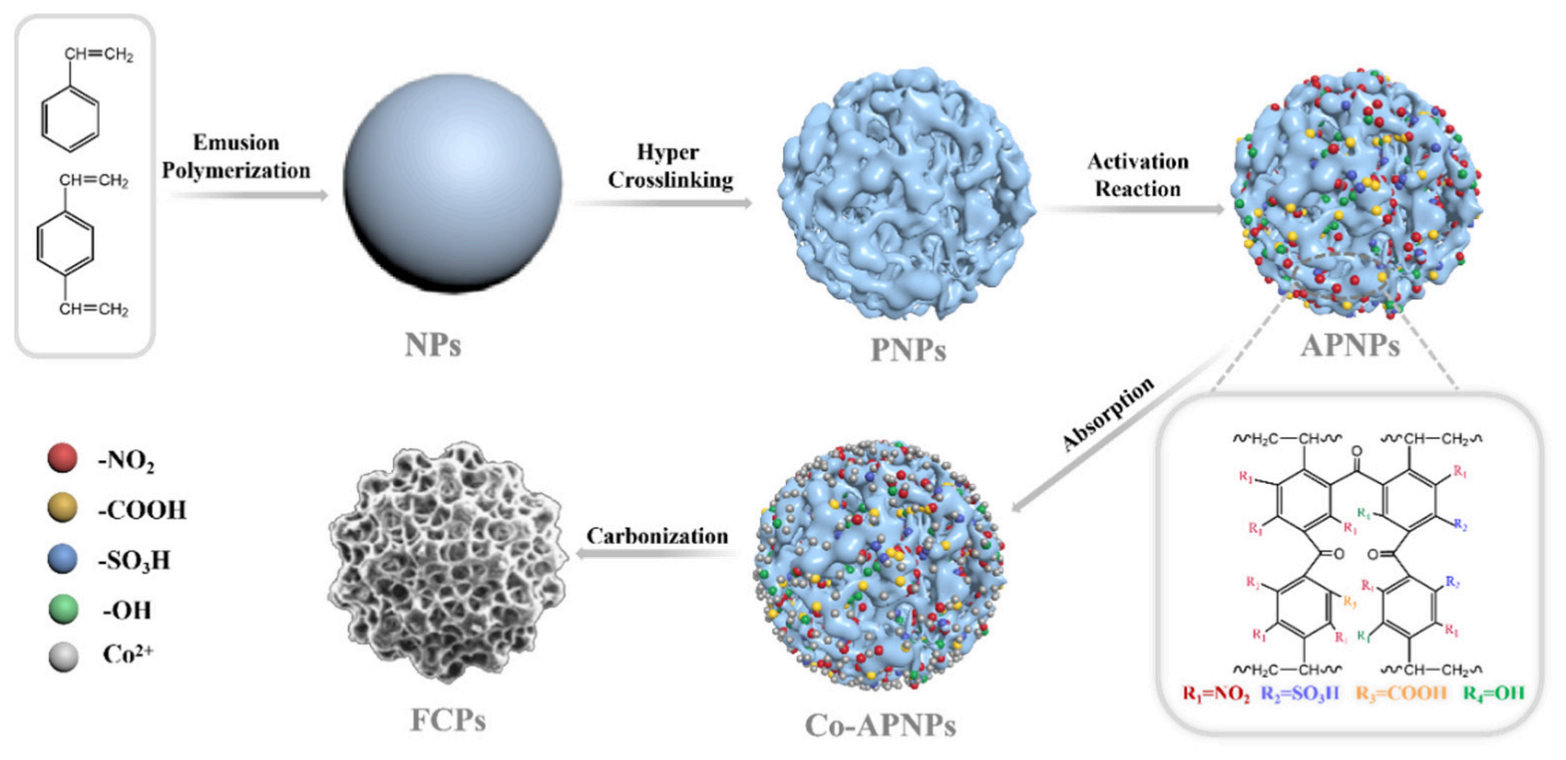
Schematic illustration for the fabrication of polystyrene-based functional carbon materials.

In this work, a synthetic strategy consisting of pre-crosslinking, Friedel-Crafts hypercrosslinking and sulfonation modification is proposed. Firstly, pre-crosslinked polystyrene nanoparticles are synthesized via emulsion polymerization using styrene and divinylbenzene as monomers. Subsequently, the Friedel-Crafts hypercrosslinking reaction is carried out to construct a rigid three-dimensional network architecture, which greatly improves the thermal stability and morphological retention capability of the precursor. On this basis, sulfonation modification is performed using mixed sulfuric and nitric acids, and abundant sulfonic acid groups are successfully grafted onto the microsphere surface, which provides an ideal platform for the efficient anchoring of metal ions and catalytic mass transfer in the subsequent procedures. Benefiting from the strong adsorption and ion-exchange capacity of sulfonic acid groups, transition metal ions including Co, Ni and Fe are doped onto the microsphere surface. After carbonization under an air atmosphere, the polymeric skeleton is converted into a carbon matrix, while metal species are in situ transformed into metal oxide nanoparticles. Meanwhile, homogeneous dispersion and stable immobilization of metallic active sites within the carbon substrate are simultaneously achieved. The design idea is shown in Figure 1.

## 2. Results and Discussion

### 2.1. Kinetics of Polymerization and Crosslinking Reactions of Styrene

This section firstly elaborates the kinetic laws governing styrene (St) polymerization. The experiments were carried out under fixed conditions: the mass ratio of styrene to divinylbenzene was set as 3:1, the deionized water dosage was 100 mL, the reaction duration was 5 h, and the reaction temperature was maintained at 75 °C, to explore how SDS addition affects the yield of polystyrene nanoparticles (NPs).

**Figure 2:**
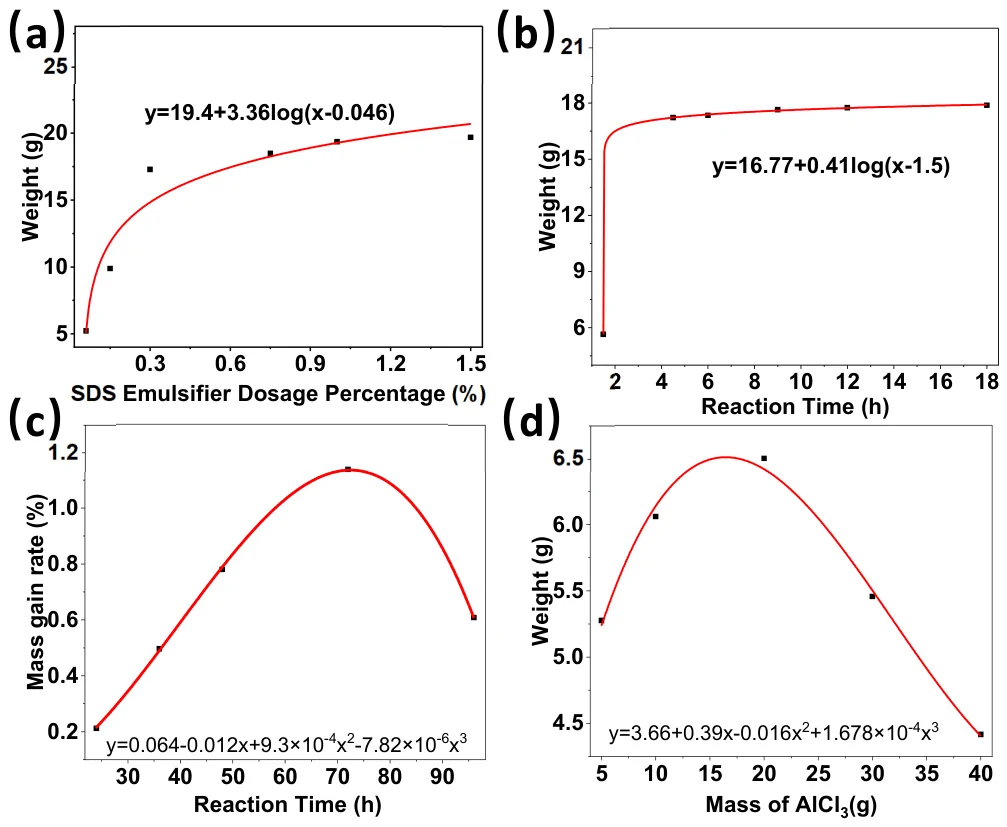
(**a**) Effect of SDS emulsifier dosage on the yield of NPs; (**b**) Influence of polymerization time on the yield of NPs; (**c**) Effect of NPs swelling time on the weight gain rate of PNPs; (**d**) Relationship between the dosage of AlCl_3_ catalyst and PNPs yield (g).

Figure 2a illustrates the influence of sodium dodecyl sulfate (SDS, emulsifier) dosage on the yield of polystyrene (PS) nanoparticles. At the initial stage, the product yield rises sharply. Once the SDS dosage reaches a certain threshold, the emulsion system achieves thermodynamic stability; further increasing the emulsifier dosage barely contributes to yield improvement, and the curve consequently enters a plateau region. Experimental results reveal that when the SDS concentration is lower than its critical micelle concentration (CMC, theoretically ranging from 0.17 wt% to 0.3 wt%, *w/v*) [32,33], the yield increases rapidly with growing SDS content. Nevertheless, when the emulsifier dosage exceeds 0.6 wt%, the number of micelle nucleation sites becomes saturated, and the growth rate of yield slows down remarkably, which conforms to the logarithmic curve described by the fitted kinetic equation.

Figure 2b depicts the effect of polymerization time on polystyrene microsphere yield. With the monomer ratio, solvent dosage, emulsifier amount and reaction temperature kept constant, the time-dependent yield curve follows a logarithmic fitting profile, consistent with the characteristic equation for chain-growth polymerization. Accordingly, the polymerization equilibrium time should be longer than 4 h.

Friedel-Crafts crosslinking serves as the pivotal procedure to enhance the thermal stability of NPs; hence, the crosslinking reaction conditions were systematically optimized in this work. Figure 2c shows the relationship between swelling time and the weight gain rate of crosslinked polystyrene nanoparticles (PNPs). The tests were performed at a fixed mass ratio of NPs to aluminum trichloride (AlCl_3_) of 1:4 and a fixed mass ratio of NPs to carbon tetrachloride (CCl_4_, crosslinker) of 1:30, with swelling time varied from 24 h to 96 h. It can be observed that the curve possesses an optimal point at a swelling time of 72 h.

Figure 2d presents the correlation between catalyst AlCl_3_ dosage and PNPs yield. Under the fixed NPs/CCl_4_ ratio of 1:30 and a reaction time of 72 h, the variation in PNPs yield was recorded as AlCl_3_ dosage increased from 5 g to 40 g. The yield rises first and then declines with increasing catalyst loading, peaking at an AlCl_3_ dosage of 20 g. When the catalyst dosage is only 5 g, insufficient aromatic ring activation leads to incomplete crosslinking reactions. As more AlCl_3_ is added, the reactive sites are fully activated, facilitating tighter crosslinking networks and elevating the product yield. However, excessive catalyst dosage triggers undesired side reactions, which disrupt the pre-formed crosslinked architectures and ultimately cause a reduction in yield.

### 2.2. Morphology and Particle Size Distribution of NPs and PNPs

**Figure 3:**
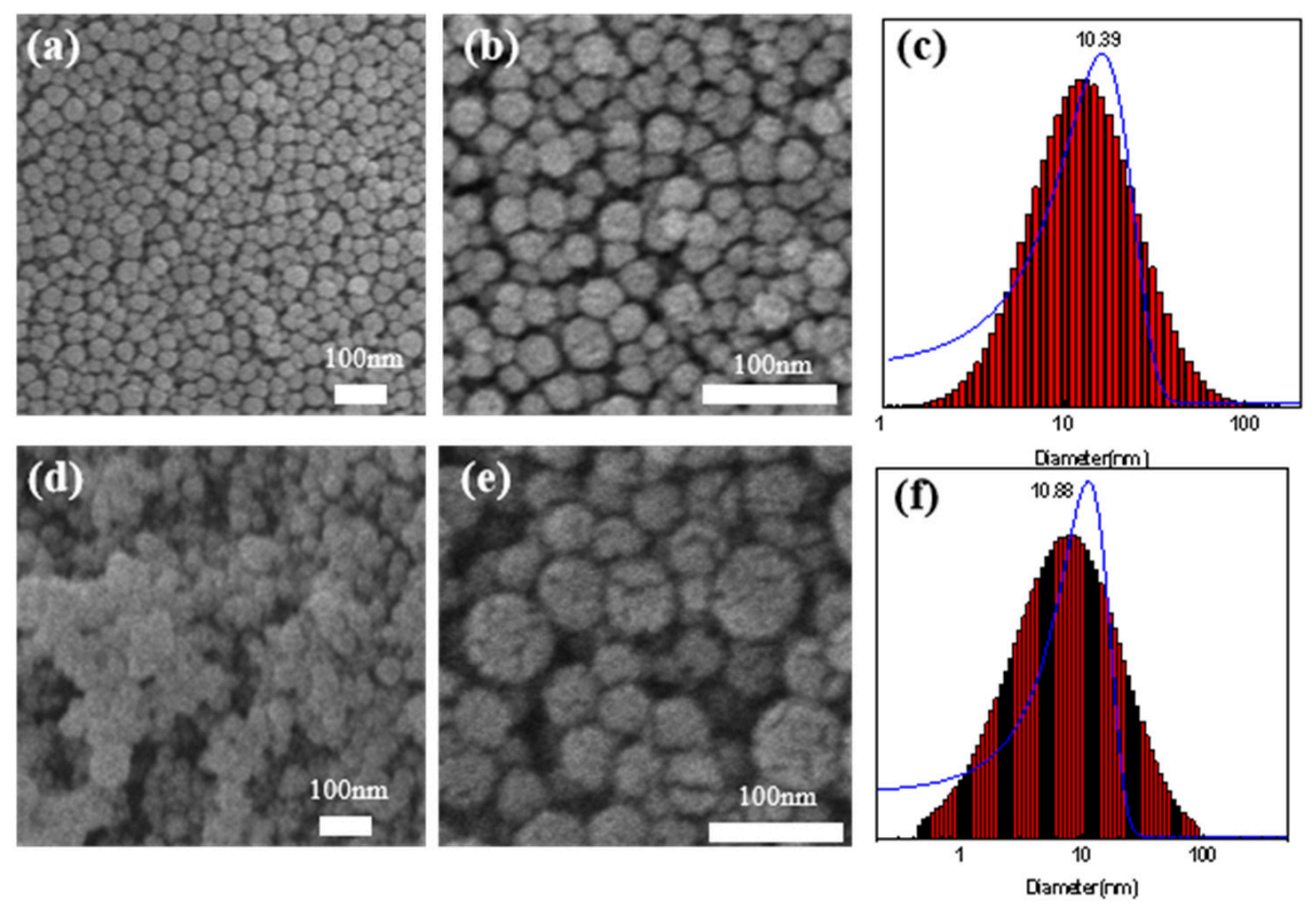
(**a**,**b**) SEM images of NPs; (**c**) particle size distribution curve of NPs; (**d**,**e**) SEM images of PNPs; (**f**) particle size distribution curve of PNPs.

The morphology and structural evolution of NPs were characterized via scanning electron microscopy (SEM). In Figure 3a, the pristine NPs exhibit regular spherical shapes with favorable dispersity and negligible agglomeration. The high-magnification SEM image (Figure 3b) reveals smooth surfaces and distinct edges of the nanoparticles, accompanied by a narrow particle size distribution. The corresponding particle size distribution curve (Figure 3c) further verifies that the particle diameters of NPs are mainly centered at approximately 10.39 nm with a concentrated distribution, which confirms their excellent dispersion performance.

After the Friedel-Crafts crosslinking reaction, the PNPs perfectly retain the overall spherical geometry (Figure 3d). Compared with pristine NPs, the surfaces of PNPs (Figure 3e) become slightly roughened. This subtle morphological variation demonstrates that the Friedel-Crafts alkylation reaction successfully constructs rigid covalent crosslinking networks among polymer chains, which densifies the material framework. This structural feature lays a crucial foundation for the precursor to maintain morphological integrity during the subsequent high-temperature carbonization procedure. The particle size distribution profile (Figure 3f) illustrates that PNPs possess a nearly identical particle size distribution to NPs, indicating that the crosslinking process barely alters the overall dimensions of nanoparticles; the average particle size of PNPs is concentrated at roughly 10.88 nm.

### 2.3. Fourier-Transform Infrared Spectra and Thermal Stability of NPs, PNPs and Activated Hypercrosslinked Polystyrene Nanoparticles (APNPs)

**Figure 4:**
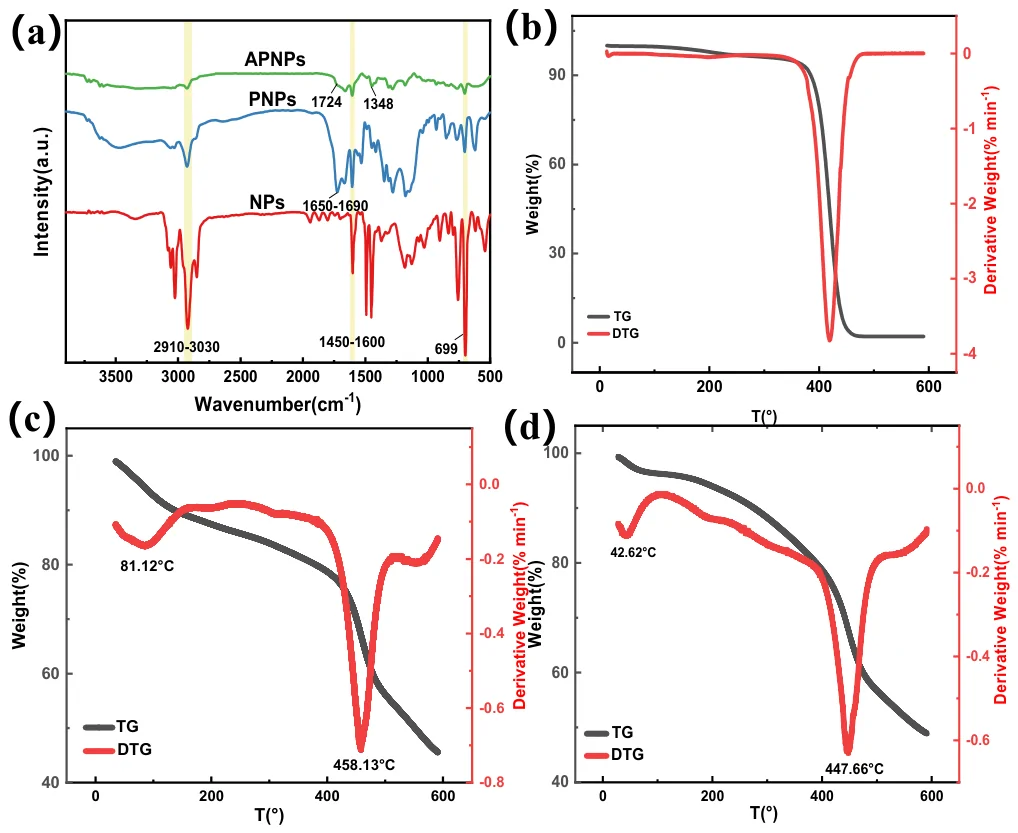
(**a**) FTIR spectra of NPs, PNPs and APNPs; (**b**–**d**) Thermogravimetric curves and corresponding derivative thermogravimetric curves of NPs, PNPs and APNPs, respectively.

Figure 4a presents the FTIR spectra of NPs, PNPs and APNPs. All samples exhibit absorption peaks at 3028 cm^−1^ and 2920 cm^−1^, which are assigned to the C-H stretching vibration of benzene rings and the asymmetric stretching vibration of methylene groups (-CH_2_-), respectively; these are intrinsic characteristic peaks of the polystyrene backbone. Multiple absorption bands ranging from 1450 cm^−1^ to 1600 cm^−1^ originate from the skeletal vibration (C=C) of aromatic rings, while the intense peaks at 699 cm^−1^ and 755 cm^−1^ correspond to the out-of-plane bending vibration of monosubstituted benzene rings. Collectively, these peaks verify the existence of the polystyrene framework. Compared with NPs, the FTIR spectrum of PNPs displays a new weak shoulder peak between 1650 cm^−1^ and 1690 cm^−1^, which may be attributed to the stretching vibration of carbonyl groups (C=O) [34]. The emergence of this peak confirms that carbonyl bridging structures are introduced during the Friedel-Crafts crosslinking reaction. After sulfonation activation, APNPs show a new broad and strong absorption band centered at approximately 1348 cm^−1^, originating from the asymmetric stretching vibration of O=S=O bonds within sulfonic acid groups (-SO_3_H). These spectral variations serve as direct spectroscopic evidence that sulfonic acid groups have been successfully grafted onto the aromatic rings of polystyrene.

Figure 4b–d show the thermogravimetric (TG) curves and derivative thermogravimetric (DTG) curves of NPs, PNPs and APNPs, respectively. The measurement was performed in an air atmosphere with a heating rate of 5 °C min^−1^. The TG profile of pristine NPs undergoes a typical drastic one-step thermal decomposition. The sample mass remains nearly unchanged below around 300 °C, demonstrating excellent structural stability within this temperature range. Mass loss initiates rapidly when the temperature rises to roughly 380 °C, and the maximum mass loss rate is achieved at approximately 420 °C. This stage corresponds to the scission and depolymerization of the polystyrene main chain, accompanied by the massive release of volatile small molecules such as styrene monomers. The residual mass of NPs decreases to less than 2 wt% at 500 °C, and nearly complete weight loss is observed at 600 °C. In contrast, PNPs exhibit a gradual and continuous weight loss process, with the maximum decomposition rate on the DTG curve occurring at about 420 °C. Notably, PNPs retain approximately 50 wt% of their initial mass at 600 °C. Such greatly enhanced thermal resistance stems from the rigid three-dimensional network constructed via Friedel-Crafts hypercrosslinking.

The TG curve of activated hypercrosslinked polystyrene nanoparticles (APNPs) features an obvious weight loss step near 250 °C, with a corresponding decomposition peak at around 260 °C on the DTG curve. This phenomenon is ascribed to the thermal decomposition of grafted sulfonic acid groups (-SO_3_H), including desulfurization and the release of sulfur dioxide (SO_2_). Afterwards, APNPs undergo a second major weight loss stage from 300 °C to 500 °C, which arises from the further decomposition of the crosslinked polymer skeleton. Owing to the prior decomposition of sulfonic acid groups and the catalytic carbonization effect induced by their strong acidity on the carbon framework, APNPs display a total weight loss of roughly 40 wt% at 600 °C. This value is slightly lower than that of PNPs but far higher than that of the original NPs.

### 2.4. The Pore Structure Analysis of PNPs

**Figure 5:**
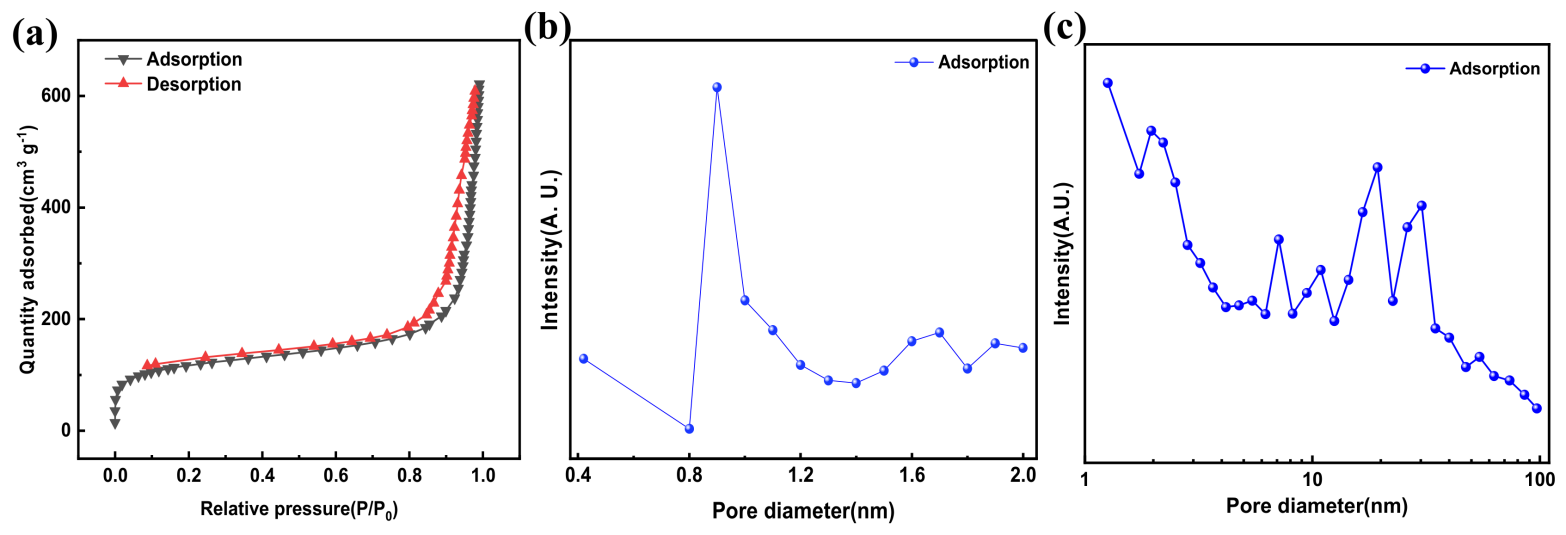
(**a**) Nitrogen adsorption–desorption isotherms of PNPs; (**b**) Micropore size distribution curve; (**c**) Mesopore size distribution curve.

The nitrogen adsorption–desorption isotherms of PNPs (Figure 5a) display a typical Type IV isotherm accompanied by a distinct H4 hysteresis loop. The sharp increase in adsorbed volume at a relative pressure (P/P_0_) below 0.1 confirms the abundant presence of micropores (<2 nm) within the material. In the medium-to-high relative pressure range of 0.4–1.0, the adsorption and desorption branches diverge to form an obvious hysteresis loop, which is a hallmark of capillary condensation within mesopores (2–50 nm) and verifies the well-developed mesoporous architecture of PNPs. Calculations reveal that the Brunauer—Emmett—Teller (BET) specific surface area of PNPs reaches 424.64 m^2^ g^−1^. The total pore volume of the sample is 0.9553 cm^3^ g^−1^. The micropore volume is 0.1434 cm^3^ g^−1^ obtained by the MP method; the mesopore volume is 0.8282 cm^3^ g^−1^ obtained from the BJH adsorption branch. The mesopores dominate the pore-volume contribution, while the contribution of micropores is relatively small.

The corresponding pore size distribution curve (Figure 5b) further clarifies the pore structural features of PNPs: the pore system is dominated by micropores with a minor proportion of mesopores. Two prominent microporous peaks are centered at approximately 1.0 nm and 1.5–2.0 nm. Meanwhile, a certain pore volume is distributed across the 2–5 nm mesoporous range with a broad pore size distribution, indicative of the inhomogeneous mesoporous structure. All results demonstrate that the crosslinked polystyrene nanoparticles possess a hierarchical porous structure with coexisting micropores and mesopores.

### 2.5. Optimization of Sulfonation Conditions

PNPs were modified via sulfonation, which may provide cation-exchange sites and could contribute to the loading of various metal ions. As presented by the elemental analysis results in Table 1, the carbon and hydrogen contents of NPs are 91.23 wt% and 7.68 wt%, respectively, which are consistent with the theoretical elemental composition of polystyrene. After Friedel-Crafts crosslinking, the carbon and hydrogen contents of PNPs decreased to 63.20 wt% and 5.24 wt%, while the oxygen content increased to 5.69 wt%, suggesting a plausible explanation for the introduction of oxygen-containing functional groups during the crosslinking reaction.

Upon sulfonation and activation, the elemental composition of APNPs changed drastically. The sample contained 8.57 wt% sulfur, 1.05 wt% nitrogen, along with an oxygen content of 16.30 wt%. The detectable sulfur element offers one plausible yet not exclusive interpretation for the potential grafting of sulfonic acid groups.

As shown in Table 2, when the dosage of sulfuric acid was increased from 10 mL to 60 mL, the sulfur content rose markedly from 2.62 wt% to 8.57 wt%. This directly verifies that increasing the sulfuric acid dosage can effectively improve the grafting density of sulfonic acid groups (-SO_3_H) anchored on the polymer framework.

Based on the sulfur content (2.62–8.57 wt%) determined by elemental analysis, the sulfonation degree of polystyrene nanoparticles can be further calculated to range from 8.7% to 30%. Assuming each sulfonic acid group (-SO_3_H) contains one sulfur atom, the molecular weight of each styrene unit (C_8_H_8_) is 104.15, and the molecular weight of each sulfonic acid group (-SO_3_H) is 81.07; the sulfonation degree (the proportion of benzene rings substituted by -SO_3_H groups) can be estimated via the following formula:

x=ws32.06(1−ws)104.15

where *x* stands for the sulfonation degree; *w_s_* is the mass fraction of sulfur measured by elemental analysis; 32.06 g mol^−1^ refers to the molar mass of elemental sulfur; (1 − *w_s_*) represents the mass fraction of the residual polymer framework excluding sulfur.

This calculation result is consistent with data reported in the previous literature and nearly reaches the maximum limit achievable via sulfonation with sulfuric acid.

### 2.6. Optimization of Carbonization Conditions

To determine the optimal carbonization process conditions, the effects of carbonization temperature and time on the yield of the samples were first investigated. As shown in Table 3, when the carbonization temperature increased from 200 °C to 600 °C, the sample yield decreased from 89.67% to 6.67%. As shown in Table 4, the effect of carbonization time on the yield was further investigated at 300 °C. When the carbonization time increased from 1 h to 9 h, the sample yield decreased from 78% to 51%.

**Figure 6:**
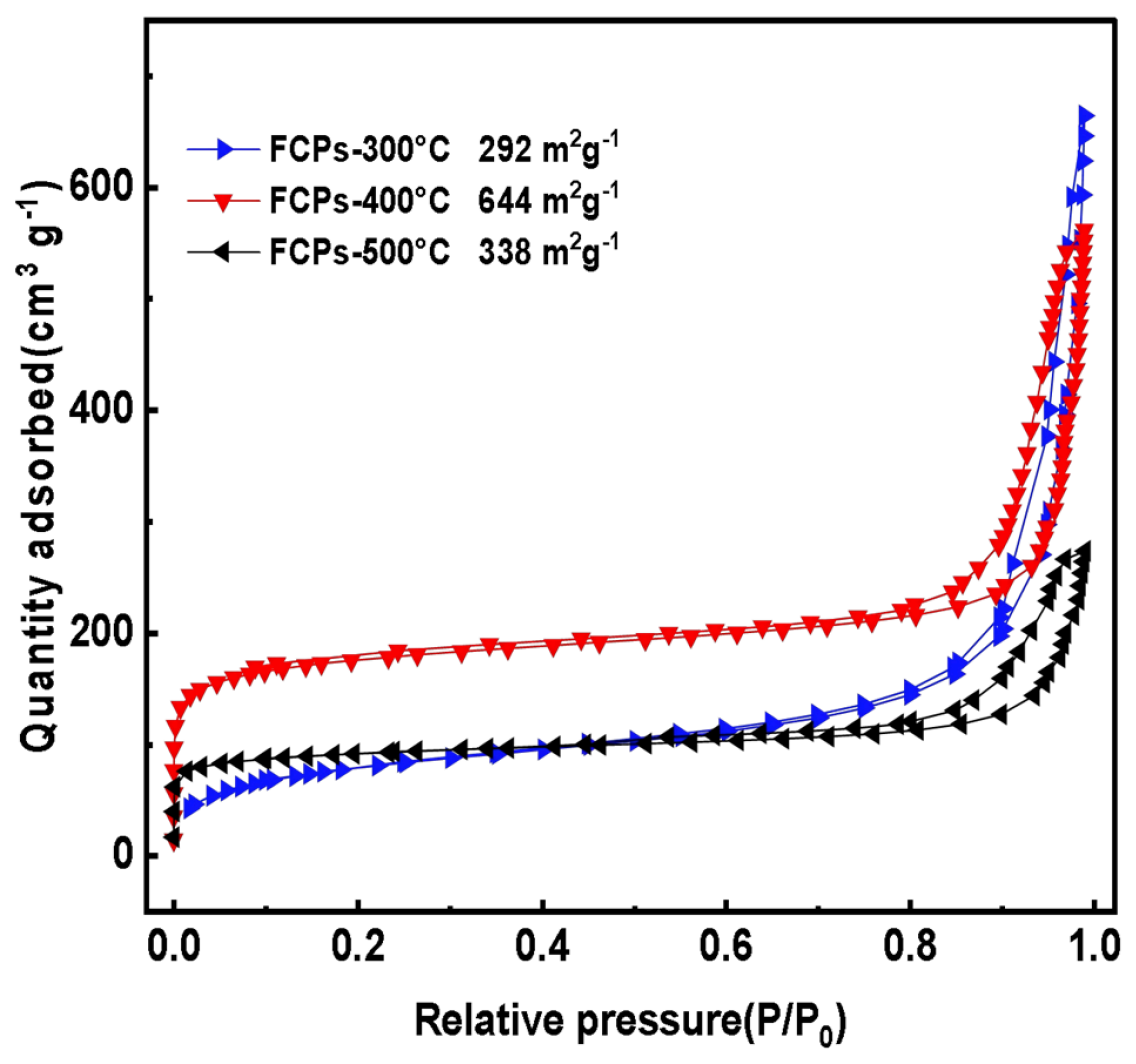
Nitrogen adsorption–desorption isotherms and specific surface areas of FCPs prepared at different carbonization temperatures.

Nitrogen adsorption–desorption measurements were conducted to analyze the pore structure of functional carbon particles (FCPs) synthesized at various temperatures, aiming to clarify the effect of carbonization temperature on the pore characteristics of functional carbon materials. The corresponding results are displayed in Figure 6.

The calculated specific surface area exhibits an obvious dependence on carbonization temperature. When the temperature was 300 °C, the specific surface area slightly increased to 292.10 m^2^·g^−1^; the maximum value of 644.06 m^2^·g^−1^ was achieved at 400 °C; further increasing the temperature to 500 °C led to a decline in specific surface area to 338.36 m^2^·g^−1^. At 300 °C, sulfonic acid groups anchored on the precursor surface underwent thermal decomposition and released small gaseous molecules such as sulfur dioxide (SO_2_), which gradually created initial pores. Nevertheless, the polymeric framework was not fully carbonized at this temperature, resulting in inadequate pore development and only a modest rise in specific surface area. Upon heating up to 400 °C, sulfonic acid groups were completely decomposed, accompanied by deep carbonization of the polymer backbone. Massive gas emission during decomposition generated abundant micropores and mesopores within the carbon matrix, thus greatly boosting the specific surface area. However, continuous temperature elevation to 500 °C triggered shrinkage and densification of the carbon skeleton. Partial micropores collapsed or merged under high temperature, which ultimately reduced the specific surface area. With the increase of carbonization temperature from 300 °C to 500 °C, the total pore volume of FCPs decreased from 1.0198 cm^3^ g^−1^ to 0.4153 cm^3^ g^−1^. At 300 °C, the mesopore volume of 0.9564 cm^3^ g^−1^ was absolutely dominant (accounting for 94%). At 400 °C, the micropore volume increased to 0.2636 cm^3^ g^−1^, and the BET specific surface area reached the maximum value of 644 m^2^ g^−1^. Upon further heating to 500 °C, the pore structure shrank, and the total pore volume dropped to the minimum value of 0.4153 cm^3^ g^−1^.

Comprehensively considering the carbonization performance and product yield, 400 °C and 1 h were selected as the optimal carbonization conditions for subsequent experiments.

### 2.7. Effect of Metal Composition on the Pore Structure of Materials

**Figure 7:**
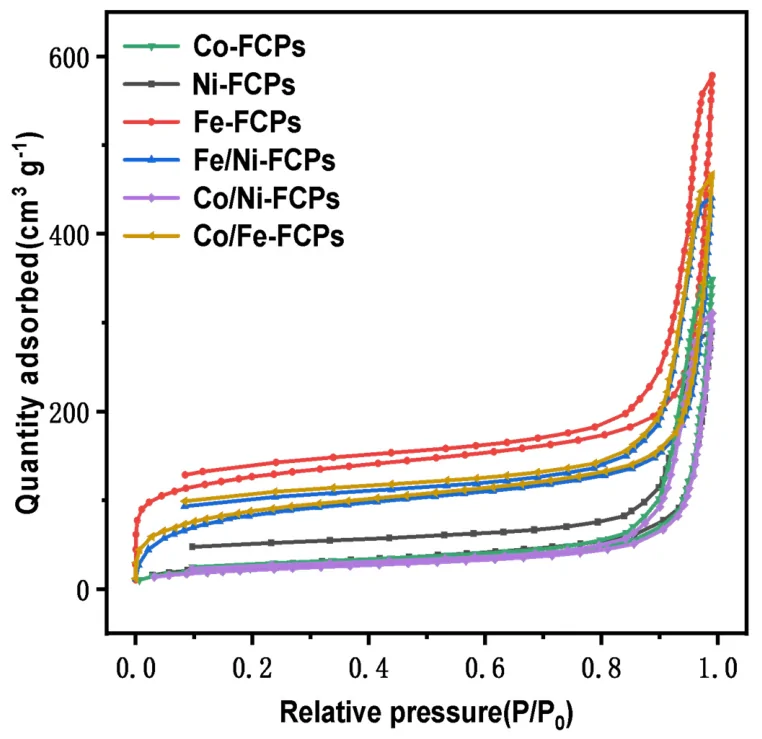
Nitrogen adsorption–desorption isotherms of Co-FCPs, Ni-FCPs, Fe-FCPs, Fe/Ni-FCPs and Co/Ni-FCPs.

To further investigate the influence of metal composition on the specific surface area and pore structure of functional carbon materials, nitrogen adsorption–desorption measurements were performed on Co-FCPs, Ni-FCPs, Fe-FCPs, Fe/Ni-FCPs, Co/Ni-FCPs and Ni-FCP samples, and the corresponding results are displayed in Figure 7. The results suggest that all materials possess abundant mesoporous structures dominated by slit-shaped pores, and such structural features are well inherited from the hierarchical porous framework of the APNPs precursor. The BET specific surface areas of Co-FCPs, Ni-FCPs and Fe-FCPs are 84.97 m^2^·g^−1^, 95.71 m^2^·g^−1^ and 455.46 m^2^·g^−1^, respectively. For bimetallic samples, the specific surface areas of Co/Ni-FCPs, Ni/Fe-FCPs and Fe/Co-FCPs are 77.50 m^2^·g^−1^, 309.58 m^2^·g^−1^ and 311.72 m^2^·g^−1^, correspondingly. The corresponding pore volumes are also listed in Table 5.

These phenomena indicate that metal species can effectively reconstruct the pore architecture of materials during the carbonization process. Notably, the catalytic performance of electrocatalysts is comprehensively determined by multiple factors, including surface chemical properties, electrical conductivity, and topological structure. The variations in specific surface area and pore structure observed in this work only reflect the changes in the microscopic structure of materials during the preparation process, which can provide a valuable reference for understanding the subsequent electrochemical application performance rather than serving as the sole evaluation criterion for catalytic activity.

### 2.8. Surface Structure Analysis of FCPs

The surface chemical composition of hybrid carbon nanoparticles exerts a direct influence on their heterogeneous catalytic performance. X-ray photoelectron spectroscopy (XPS) was employed to characterize the surface structure of the as-prepared materials, and the high-resolution spectra of each element were subjected to peak fitting and deconvolution. The peak centered at 284.8 eV in the C 1 s spectrum is assigned to C-C bonds, which may indicate the coexistence of graphitic and amorphous carbon after carbonization. Nevertheless, C 1 s spectra alone cannot unambiguously distinguish these two carbon forms [35].

**Figure 8:**
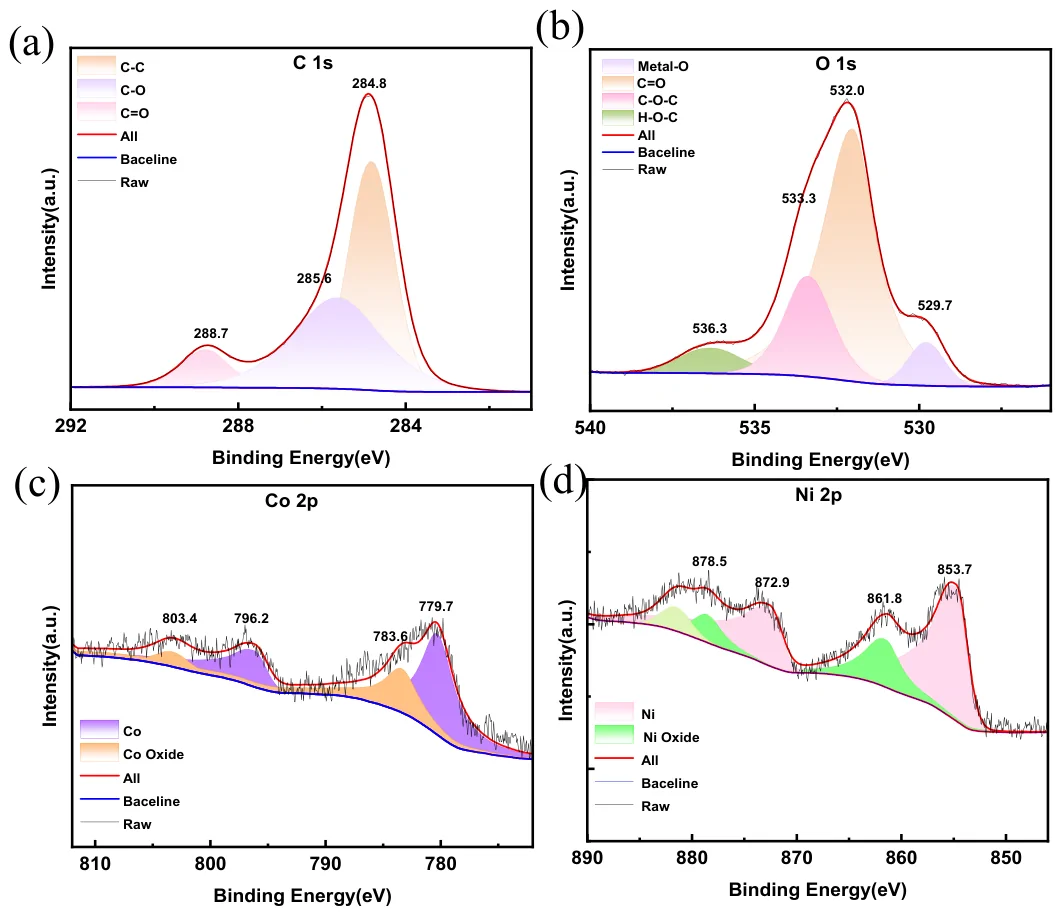
High-resolution X-ray photoelectron spectroscopy (XPS) spectra of Co/Ni-FCPs for elements C (**a**), O (**b**), Co (**c**), and Ni (**d**).

Several minor peaks located at 284.8 eV, 285.6 eV and 288.7 eV confirm the presence of C-C, C-O and C=O bonds (Figure 8a). The two peaks resolved from the high-resolution O 1s spectrum correspond to C=O bonds (532 eV) and metal-O bonds (529.7 eV), respectively (Figure 8b). For the high-resolution Co 2p spectrum, deconvolution yields two components at 779.7 eV and 776.2 eV, suggesting multiple chemical environments for cobalt-containing species on the surface (Figure 8c). The high-resolution Ni 2p spectra show a similar trend, further demonstrating the presence of metal-oxygen interactions for surface-bound Co and Ni species (Figure 8d).

Collectively, these XPS observations imply that metal-oxide-related species may contribute to the active sites toward the oxygen evolution reaction (OER). As a plausible mechanism, sulfonic acid groups are presumed to only anchor and disperse metal species, while sulfur species would scarcely remain upon subsequent carbonization under an air atmosphere.

### 2.9. Electrochemical Performances of FCPs

**Figure 9:**
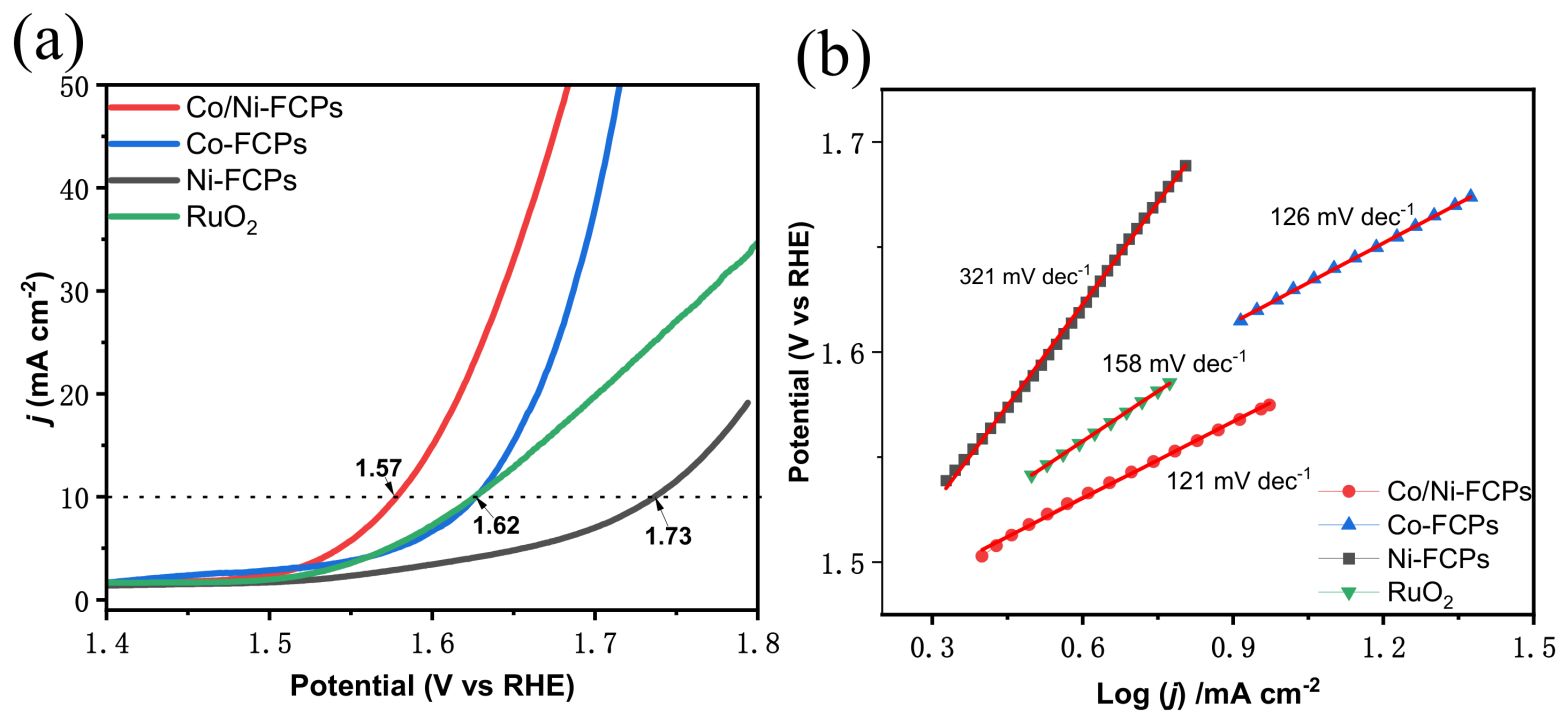
(**a**) OER polarization curves and (**b**) corresponding Tafel plots of Co/Ni-FCPs, Co-FCPs, Ni-FCPs and RuO_2_ catalysts.

Linear sweep voltammetry (LSV) polarization curves were employed to evaluate the OER electrocatalytic activity of the as-prepared catalysts. (Figure 9a) At the benchmark current density of 10 mA cm^−2^, Co/Ni-FCPs only require a potential of 1.57 V vs. RHE, outperforming Co-FCPs (1.62 V vs. RHE), benchmark RuO_2_ (1.62 V vs. RHE) and Ni-FCPs (1.73 V vs. RHE). Monometallic Ni-FCPs exhibit the poorest OER activity. The introduction of Co improves catalytic performance, and the Co-Ni composite further reduces the operating potential, indicating the positive synergistic effect between Co and Ni. Co/Ni-FCPs also deliver the highest current response at high current densities, demonstrating outstanding OER catalytic performance.

Tafel plots were employed to evaluate the OER kinetics of the catalysts. (Figure 9b) The Co/Ni-FCPs present a Tafel slope of 121 mV dec^−1^, which is slightly lower than that of Co-FCPs (126 mV dec^−1^) and far superior to Ni-FCPs (321 mV dec^−1^), indicating that the combination of Co and Ni can effectively accelerate the OER kinetics. Under identical test conditions, both Co/Ni-FCPs and Co-FCPs possess smaller Tafel slopes than the benchmark RuO_2_ (158 mV dec^−1^), further verifying the excellent electrocatalytic kinetics of the composite catalyst. The relatively high apparent potential and large Tafel-slope observed for the RuO_2_ benchmark in this work can be largely ascribed to the lack of iR compensation across all electrochemical measurements.

**Figure 10:**
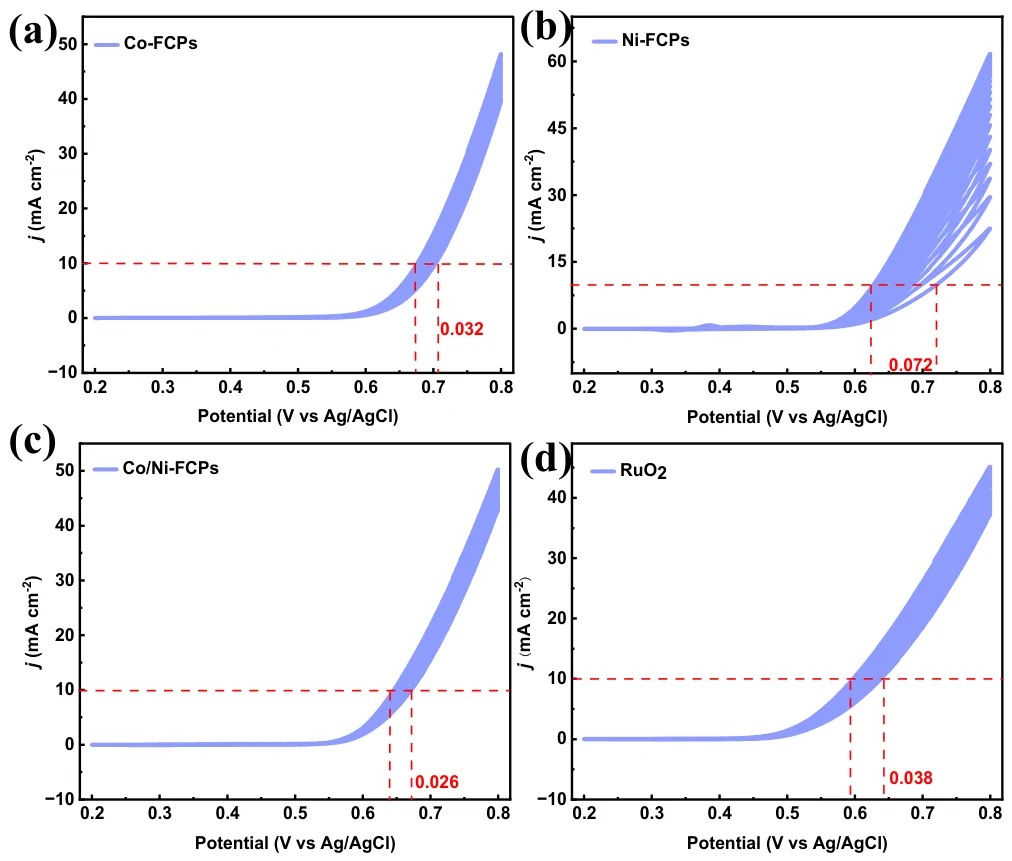
Cyclic stability CV curves of Co-FCPs (**a**), Ni-FCPs (**b**), Co/Ni-FCPs (**c**) and RuO_2_ (**d**) after 50 cycles.

After systematically evaluating the oxygen evolution reaction (OER) catalytic activities of Co-FCPs, Ni-FCPs and Co/Ni-FCPs, cyclic voltammetry (CV) measurements were implemented to investigate their electrochemical stability, and the results are displayed in Figure 10. The polarization curve variations before and after 50 consecutive CV cycles are compared among the three functional carbon materials and the commercial RuO_2_ catalyst.

After 50 cycles at a current density of 10 mA·cm^−2^, the voltage fluctuations of Co-FCPs, Ni-FCPs and Co/Ni-FCPs were 0.032 V, 0.072 V and 0.026 V, respectively. Both single-metal catalysts (Co-FCPs and Ni-FCPs) suffered from catalytic performance degradation. This phenomenon is presumed to originate from the agglomeration of metallic nanoparticles or the leaching of surface-active species during repeated electrochemical cycling. By contrast, Co/Ni-FCPs only presented a tiny voltage fluctuation of 0.026 V after 50 cycles, and its polarization curve nearly overlapped with the initial profile. Unexpectedly, commercial RuO_2_ delivered a larger voltage variation at the identical current density.

The excellent cycling stability of Co/Ni-FCPs is likely associated with the possible synergistic effect between bimetallic components. The electronic interaction between Co and Ni may optimize the electronic configuration of catalytic active sites and could strengthen metal-oxygen bonding, which potentially alleviates the dissolution of active species during electrocatalysis. Furthermore, the relatively strong interfacial coupling between bimetallic species and carbon support may restrict the migration and agglomeration of metal nanoparticles during long-term cycling. In addition, the hierarchical porous structure of the carbon matrix could provide a stable microenvironment for active sites, which favors the maintenance of catalytic stability.

## 3. Materials and Methods

### 3.1. Preparation of Polystyrene-Based Precursors

Appropriate amounts of sodium dodecyl sulfate (SDS) were dispersed in deionized water contained in a three-necked flask, followed by continuous mechanical stirring to achieve homogeneous dispersion of SDS in the aqueous system; meanwhile, the system was heated to 50 °C via a water bath. Subsequently, a mixed monomer system consisting of styrene (St) and divinylbenzene (DVB) with a mass ratio of 3:1 was added into the flask, and the temperature was raised to 75 °C for emulsification treatment. Afterwards, a certain dosage of potassium persulfate (K_2_S_2_O_8_) was dissolved in 10 mL deionized water under heating until fully dissolved, and the obtained initiator solution was poured into the reaction mixture. The polymerization reaction was maintained at a constant temperature for a predetermined duration. After the reaction was completed, the system was cooled down and dried to obtain pristine polystyrene nanoparticles (denoted as NPs).

Friedel-Crafts crosslinking was performed to construct a rigid framework of carbon precursors from crosslinked polystyrene. Briefly, polystyrene nanoparticles (NPs) were immersed in carbon tetrachloride for sufficient swelling and dispersion for a set period. The mixture was heated to 75 °C, aluminum chloride (AlCl_3_) was added as the Lewis acid catalyst, and the crosslinking reaction proceeded for 24 h. The reaction was quenched by adding deionized water for hydrolysis. After hydrolysis, the product was collected by vacuum filtration to yield hypercrosslinked polystyrene nanoparticles (PNPs).

A predetermined mass of PNPs was mixed with concentrated sulfuric acid and nitric acid in a reaction vessel, and sulfonation was carried out at a designated temperature for a certain time. Upon completion of the sulfonation reaction, the mixture was diluted, pH-adjusted, repeatedly washed with deionized water, and fully dried. The final surface-activated hypercrosslinked polystyrene nanoparticles were designated as APNPs.

### 3.2. Fabrication of Polystyrene-Derived Functional Carbon Materials

A given mass of each precursor powder was transferred into a porcelain crucible, heated from room temperature to 300–600 °C at a heating rate of 1 °C min^−1^, and maintained at the target temperature for 1–9 h. By comprehensively evaluating carbonization behavior and product yield, 400 °C with a holding time of 1 h was determined as the optimal carbonization condition for subsequent experiments, and these parameters were fixed for all further carbonization procedures.

Prior to carbonization, APNPs were separately mixed with aqueous solutions of cobalt(II) nitrate hexahydrate, iron(III) nitrate nonahydrate, and nickel hydroxide (dissolved with an appropriate amount of hydrochloric acid) in beakers. Each mixture was magnetically stirred at room temperature for 24 h to achieve adequate metal-ion adsorption. After stirring, the obtained slurries were dried in an oven. The dried solids were thoroughly ground in an agate mortar to yield metal-adsorbed precursor samples. These metal-loaded precursors were then carbonized under the above-mentioned optimal conditions to obtain functional porous carbon nanoparticles, which are abbreviated as FCPs herein. Samples loaded with monometallic cobalt, iron, and nickel are denoted as Co-FCPs, Fe-FCPs, and Ni-FCPs, respectively. Bimetallic Co/Ni-doped carbon materials were also prepared following the identical synthetic procedure and designated as Co/Ni-FCPs.

### 3.3. Nitrogen Adsorption–Desorption Isotherms and Data Processing Methods

The samples were degassed under vacuum at 105 °C for 4 h to remove adsorbed moisture and surface impurities. The degassed sample cell was then transferred to the instrument test chamber for the measurement of low-temperature nitrogen adsorption–desorption isotherms. The specific surface area was calculated using the Brunauer—Emmett—Teller (BET) model. The MP method and Barrett—Joyner—Halenda (BJH) model were employed to analyze the pore-size distribution and pore volume over the full pore-size range, enabling accurate characterization of the full-range pore-structure parameters.

### 3.4. Oxygen Evolution Reaction (OER)

Electrochemical measurements of the as-prepared samples were carried out using a three-electrode configuration. An Ag/AgCl electrode served as the reference electrode, a graphite rod (the platinum sheet was only used for LSV measurements) was used as the counter electrode, and a glassy carbon electrode loaded with the catalytic active material acted as the working electrode. All percentages given below refer to mass fractions. Catalyst inks were prepared using an identical formulation consisting of 70 wt% active catalyst (2 mg), 10 wt% Nafion binder, and 20 wt% conductive carbon black, dispersed in a mixed ethanol-water solvent (250 μL of absolute ethanol and 200 μL of deionized water). A 10 μL aliquot of the resultant dispersion was drop-cast onto the glassy carbon electrode surface (geometric area: 0.1963 cm^2^), followed by natural evaporation to dryness under ambient conditions. Similarly, the RuO_2_ benchmark electrode employed the same ink composition: 70 wt% RuO_2_, 20 wt% conductive carbon black, and 10 wt% Nafion binder, with the same ethanol-water solvent volume. Aqueous KOH solution with a concentration of 1 mol L^−1^ was employed as the electrolyte throughout the tests. Linear sweep voltammetry (LSV) tests were performed at a scan rate of 10 mV s^−1^ with a voltage window ranging from 0.2 V to 0.8 V.

All potentials recorded against the Ag/AgCl reference electrode were converted to the reversible hydrogen electrode (RHE) scale for polarization curve plotting according to the following equations:

E (V vs. RHE) = E′ (Ag/AgCl) + 0.0591 × pH + 0.197

*J* = *I*/*S*

where E′(Ag/AgCl) is the measured potential referenced to the Ag/AgCl electrode; *I* represents the measured current; S stands for the geometric area of the working electrode (0.1963 cm^2^); *J* denotes the current density; *pH* refers to the pH value of the alkaline electrolyte. The constant of 0.197 V corresponds to the standard potential of the saturated Ag/AgCl electrode at 25 °C.

The OER overpotential (*η*) was defined as *η* = E_RHE_ − 1.23 V, in which 1.23 V represents the thermodynamic equilibrium potential for the oxygen evolution reaction. The potential vs. RHE was converted from experimental potentials recorded with an Ag/AgCl reference electrode. No iR compensation was applied to the polarization curves.

### 3.5. Cycling Stability Measurements

Cyclic voltammetry (CV) tests were performed on a CHI660E electrochemical workstation with the aforementioned three-electrode system. The electrolyte was a 1 mol L^−1^ KOH solution saturated with oxygen. The reference electrode was Ag/AgCl, the counter electrode was a carbon rod, and a carbon plate with a loading area of 1 cm × 1 cm was adopted as the working electrode. The CV scanning potential window was set between 0.2 V and 0.8 V, with a scan rate of 10 mV s^−1^. The cyclic scanning was conducted for 50 cycles, with an interval of 10 s between each cycle.

## 4. Conclusions

This study systematically investigates the design, fabrication and functionalization of polystyrene-based functional carbon nanoparticles. Precursor APNPs with abundant sulfonic acid anchoring sites and greatly enhanced thermal stability are synthesized via emulsion polymerization, Friedel-Crafts hypercrosslinking and sulfonation activation. APNPs are adopted as supports to load single or bimetallic species including Co, Ni and Fe, which are further carbonized under an air atmosphere to obtain hierarchically porous carbon materials, all of which exhibit Type IV nitrogen adsorption isotherms with H4 mesoporous hysteresis loops. After 50 cyclic voltammetry cycles, Co/Ni-FCPs exhibit only a minor voltage fluctuation of 0.026 V, and their polarization curve nearly overlaps with the initial profile, suggesting stable performance within this limited cyclic measurement.

## Figures and Tables

**Table 1:** Elemental Contents of NPs, PNPs and APNPs Determined by Elemental Analysis.

Sample	C(wt%)	H(wt%)	N(wt%)	S(wt%)	O(wt%)
NPs	91.23	7.68	0.04	/	1.21
PNPs	63.2	5.24	0.08	/	5.69
APNPs	39.68	3.66	1.05	8.57	16.3

**Table 2:** Elemental Contents Obtained by Elemental Analysis under Different Dosages of Sulfuric Acid.

Amount of Sulfuric Acid Added (mL)	C(wt%)	H(wt%)	N(wt%)	S(wt%)	O(wt%)
10	62.34	4.85	1.4	2.62	18.71
20	56.63	4.42	1.74	3	21.4
30	52.69	4.19	1.59	4.38	21.9
60	39.68	3.66	1.05	8.57	24.63

**Table 3:** Effect of carbonization temperature on the yield of FCPs.

APNPs Weight/g	Heating Time/h	Holding Time/h	Temperature/°C	Weight/g	Yield
0.3	1	1	200	0.269	89.67%
0.3	1	1	300	0.235	78.33%
0.3	1	1	400	0.088	29.33%
0.3	1	1	500	0.048	16%
0.3	1	1	600	0.02	6.67%

**Table 4:** Effect of carbonization time on the yield of FCPs.

APNPs Weight/g	Heating Time/h	Holding Time/h	Temperature/°C	Weight/g	Yield
0.3	1	1	300	0.235	78.33%
0.3	1	2	300	0.197	65.67%
0.3	1	3	300	0.233	77.67%
0.3	1	6	300	0.157	52%
0.3	1	9	300	0.155	51.67%

**Table 5:** BET specific surface area and pore volume of different samples.

Sample	S_BET_ (m^2^·g^−1^)	Pore Volume (cm^3^·g^−1^)	Micropore Volume (cm^3^·g^−1^)
Co-FCPs	84.97	0.5200	0.0130
Ni-FCPs	95.70	0.4486	0.0162
Fe-FCPs	455.46	0.8839	0.1750
Fe/Ni-FCPs	309.58	0.6804	0.1101
Co/Ni-FCPs	77.50	0.4703	0.0088
Co/Fe-FCPs	311.72	0.7168	0.1172
Carbon black	60.80	0.1732	0.0101

## Data Availability

The original contributions presented in this study are included in the article. Further inquiries can be directed to the corresponding author.
